# Relationship between Plasma Concentrations of Afatinib and the Onset of Diarrhea in Patients with Non-Small Cell Lung Cancer

**DOI:** 10.3390/biology10101054

**Published:** 2021-10-17

**Authors:** Hayato Yokota, Kazuhiro Sato, Sho Sakamoto, Yuji Okuda, Mariko Asano, Masahide Takeda, Katsutoshi Nakayama, Masatomo Miura

**Affiliations:** 1 Department of Pharmacy, Akita University Hospital, Akita 010-8543, Japan; hayato@hos.akita-u.ac.jp; 2 Department of Internal Medicine, Division of Respiratory Medicine, Akita University School of Medicine, Akita 010-8543, Japan; kazuhiro@doc.med.akita-u.ac.jp (K.S.); ssakamoto@med.akita-u.ac.jp (S.S.); yokuda@med.akita-u.ac.jp (Y.O.); nmari@doc.med.akita-u.ac.jp (M.A.); takeda-56@hos.akita-u.ac.jp (M.T.); kat_n1@med.akita-u.ac.jp (K.N.)

**Keywords:** afatinib, diarrhea, limited sampling strategy, plasma concentration, therapeutic drug monitoring

## Abstract

**Simple Summary:**

Higher afatinib plasma concentrations have been reported to be associated with the severity of diarrhea; however, the specific target plasma concentration of afatinib required to avoid severe diarrhea onset is unclear. We found that an afatinib AUC_0–24_ of greater than or equal to 823.5 ng·h/mL and C_0_ of greater than or equal to 28.5 ng/mL may be used as cut-off values for the incidence of afatinib-induced grade 2 diarrhea. A significant correlation between the AUC_0–24_ and C_0_ of afatinib was observed (*r^2^* = 0.761; *p* < 0.001). Therefore, we could use C_0_ as a marker of therapeutic drug monitoring. In the current study, the median time to the incidence of grade 2 diarrhea in patients with a C_0_ of more than 28.5 ng/mL was 16 days. Therefore, we recommend monitoring the C_0_ of afatinib on day 8 after the beginning of afatinib therapy.

**Abstract:**

We evaluated the area under the plasma concentration–time curve (AUC) of afatinib required to avoid the onset of grade 2 or higher diarrhea. The C_0_ and AUC_0–24_ of afatinib were significant higher in patients with grade 2 diarrhea than in those with grade 0–1 diarrhea. The areas under the receiver operator curves were 0.795 with the highest sensitivity (89%) and specificity (74%) at an AUC_0–24_ threshold of 823.5 ng·h/mL, and 0.754 with the highest sensitivity (89%) and specificity (74%) at a C_0_ threshold of 28.5 ng/mL. In Kaplan–Meier analysis based on these cut-off AUC_0–24_ and C_0_ values, the median time to the incidence of grade 2 diarrhea was 16 days. The predicted AUC_0–24_ of afatinib from the single point of C_6_ showed the highest correlation with the measured AUC_0–24_ (*r^2^* = 0.840); however, a significant correlation between the AUC_0–24_ and C_0_ was also observed (*r^2^* = 0.761). C_0_ could be used as a marker of therapeutic drug monitoring because afatinib C_0_ was related to AUC_0–24_. Therefore, afatinib C_0_ should be monitored on day 8 after beginning therapy, and the daily dose of afatinib should be adjusted as an index with a cut-off value of 28.5 ng/mL.

## 1. Introduction

Afatinib is a second-generation tyrosine kinase inhibitor and irreversible ErbB-family blocker that is used for the first-line treatment of patients with epidermal growth factor receptor (EGFR) mutation-positive non-small cell lung cancer (NSCLC) [1]. Diarrhea is a common side effect associated with afatinib treatment [2,3,4,5,6,7,8], and in clinical practice, the onset of diarrhea following afatinib treatment results in temporary withdrawal or discontinuation of therapy. Among EGFR-tyrosine kinase inhibitor (TKI) treatments, afatinib causes a significantly higher rate of diarrhea than erlotinib or gefitinib [9,10,11].

In the Japanese analysis of the LUX-Lung3 clinical trial, 75.9% of patients administered afatinib therapy required a dose reduction owing to severe side effects, 22% of which were diarrhea of Common Terminology Criteria for Adverse Events (CTCAE) grade 3 [12]. Afatinib-induced diarrhea has been reported to occur in 50–62% of patients within the first 7 days of treatment and in 71% of patients within 14 days [13]. However, the mechanisms of afatinib-induced diarrhea remain poorly understood.

To date, higher afatinib plasma concentrations have been reported to be associated with the severity of diarrhea [14,15,16,17,18]. Therefore, the analysis of plasma concentrations of afatinib may enable the avoidance of diarrhea onset. However, the specific target plasma concentration of afatinib required to avoid severe diarrhea onset is not clear. The area under the plasma concentration–time curve (AUC) is generally the best parameter to indicate drug exposure, and the calculation of AUC is important for assessing the relationships between drug exposure and side effects. However, the calculation of AUC is rarely used in clinical practice because it requires multiple blood sample points, which is painful and time-consuming for patients. Therefore, the plasma trough concentration (C_0_) at pre-dose is usually used to predict efficacy or toxicity, although one point of C_0_ may not accurately indicate afatinib exposure. Limited sampling strategies (LSSs) have been proposed to overcome these difficulties. However, the LSS for predicting the AUC of afatinib has not yet been reported.

Accordingly, in this study, we calculated the target AUC_0–24_ of afatinib to avoid the onset of CTCAE grade 2 or higher diarrhea. In addition, we developed a model to predict the AUC_0–24_ of afatinib using an LSS. Subsequently, we investigated whether the predicted AUC_0–24_ of afatinib from C_0_ alone could provide an accurate approximation of the actual AUC_0–24_.

## 2. Materials and Methods

### 2.1. Patients and Protocols

Thirty-one Japanese patients with EGFR mutation-positive NSCLC (15 women and 16 men) who were hospitalized from October 2014 through December 2020 were consecutively enrolled in this study. The grade for diarrhea was determined based on CTCAE version 4.0. Three patients (2 women and 1 man) were excluded because of withdrawal due to CTCAE grade 3 diarrhea just after beginning and before blood sampling for afatinib pharmacokinetics. Patient characteristics at the start of afatinib therapy are listed in Table 1. The study protocol was approved by the Ethics Committee of Akita University School of Medicine (approval no. 790), and all patients gave written informed consent. This study was performed in accordance with the guidelines of the Declaration of Helsinki.

An initial dose of 30 or 40 mg afatinib (Giotrif; Boehringer Ingelheim, Tokyo, Japan) was orally administered once daily at a designated time (11:00 a.m.). On day 15 after beginning afatinib therapy, whole blood samples were collected just prior to (C_0_, 24 h after the 14th administration) and at 1, 2, 4, 6, 8, 12, and 24 h after the 15th administration of afatinib. Plasma was isolated by centrifugation at 1900× *g* for 15 min and was stored at −80 °C until analysis. For the 15 days prior to plasma sampling, nurses managed the administration of afatinib for hospitalized patients.

### 2.2. Analytical Methods

Plasma concentrations of afatinib were measured by high-performance liquid chromatography (HPLC) and ultraviolet methods, as previously described [19,20,21]. Following the addition of gefitinib (5 ng/10 µL methanol) as an internal standard to a 200-µL plasma sample, the plasma sample was diluted with 800 µL water and vortexed for 30 s. This mixture was applied to an Oasis hydrophilic lipophilic balance extraction cartridge (1 mL, 30 mg) that had been activated previously with methanol and water (1.0 mL each). The cartridge was then washed with 1.0 mL water and 1.0 mL of 60% methanol in water and eluted with 1.0 mL of 100% methanol. Eluates were dried by vortex-vacuum evaporation at 70 °C using a rotary evaporator (AS-ONE CVE-2AS; Osaka, Japan). The resulting residue was then dissolved in 20 µL methanol and vortexed for 30 s; 20 µL of the mobile phase was added to the sample, and the sample was vortexed for another 30 s. A 20-µL aliquot of the sample was then processed by HPLC. The calibration curve of afatinib in plasma was linear over the concentration range of 5 to 250 ng/mL. The limit of quantification of afatinib for this assay was 5 ng/mL. The coefficients of variation and accuracies for intra- and interday assays at the concentration range of 5 to 250 ng/mL were less than 12.4% and within 11.3%, respectively.

### 2.3. Pharmacokinetic Analysis

Pharmacokinetic analysis of afatinib was carried out using the standard noncompartmental method with WinNonlin (Pharsight Co., Mountain View, CA, USA; version 5.2). The total area under the observed plasma concentration–time curve (AUC) and the partial AUC from 6 to 12 h (AUC_6–12_), which are estimates of enterohepatic circulation, were calculated using the linear trapezoidal rule. The maximum plasma concentration (C_max_) and minimum plasma concentration (C_min_) of afatinib were obtained directly from the profile.

### 2.4. Statistical Analyses

The estimated glomerular filtration rate (eGFR) was calculated for each patient according to the following formula: eGFR = 194 × serum creatinine (mg/dL)^−1.094^ × age^−0.287^ × body surface area (m^2^)/1.73 (× 0.739 for women). Shapiro–Wilk tests were used to assess distributions. The clinical characteristics of patients at baseline before afatinib therapy were expressed as the number or mean value ± standard deviation (SD) (range). The Spearman’s rank correlation coefficient test was applied to assess correlations between the AUC_0–24_ of afatinib and clinical characteristics of the patient. Pharmacokinetic parameters of afatinib and the clinical characteristics of patients at the onset of diarrhea were expressed as median values (quartile 1–quartile 3). Pharmacokinetic parameters of afatinib or the clinical characteristics of patients between the two grade groups of afatinib-induced diarrhea classified by CTCAE were compared using the Mann–Whitney test. Receiver operating characteristic (ROC) curves were used to determine the best cut-off values for predictive factors, which had a minimum distance from the upper left corner to the point on the ROC curve. The Kaplan–Meier method and log-rank test were adopted to estimate and compare the cumulative incidence of grade 2 diarrhea. Multiple linear regression analysis of the AUC_0–24_ best estimates against afatinib concentrations at various time points (independent variables) was performed to develop the prediction formula for estimating individual AUC_0–24_ values. This analysis produced the following prediction formula: AUC_0–24_ = A_0_ + A_1_ × C_1_ + A_2_ × C_2_ + … + A*_n_* × C*_n_*, where A*_n_* is the coefficient and the number of samples is variable. The predictive performance of the LSS was determined by the bootstrap method [22]. We generated 1000 bootstrap samples only once to reduce the variability of results for all regression analysis methods. The distribution of the misclassification rate obtained during all bootstrap runs was used to estimate the 95% confidence interval (CI).

Results with *p*-values less than 0.05 were considered statistically significant. Statistical analyses were performed with IBM SPSS Statistics 27.0 for Windows (SPSS IBM Japan Inc., Tokyo, Japan).

## 3. Results

### 3.1. Patient Characteristics

The characteristics of patients before afatinib therapy are listed in Table 1. The mean (± SD) age of patients was 67.4 ± 7.7 years, and the means (±SDs) of body weight, body surface area, and body mass index were 57.3 ± 9.4 kg, 1.59 ± 0.16 m^2^, and 22.7 ± 1.5 kg/m^2^, respectively. There were no patients with serious renal or hepatic dysfunction before afatinib therapy. The numbers of patients with stage IV, IIIb, and IIb adenocarcinoma were 26, 1, and 1, respectively. The types of *EGFR* mutations were as follows: exon 19 deletions in 16 patients, exon 21 L858R in 7 patients, and other in 5 patients.

### 3.2. Afatinib Plasma Concentration–Time Profiles and Correlations between the AUC_0–24_ and Clinical Characteristics

**Figure 1:**
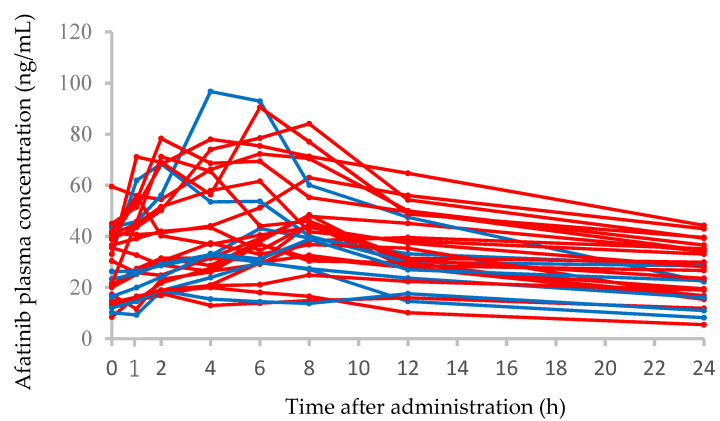
Plasma concentration–time profiles of afatinib in 28 patients administered afatinib at 30 mg/day (blue solid line) or 40 mg/day (red solid line).

Plasma concentration–time profiles from 0 to 24 h after the administration of afatinib on day 15 after the beginning of therapy in 28 patients are shown in Figure 1. The median (range) C_0_, C_max_, and AUC_0–24_ of afatinib at the steady state on day 15 in seven patients receiving 30 mg/day afatinib therapy were 23.3 (10.2–43.6) ng/mL, 38.9 (18.8–96.7) ng/mL, and 662 (357–1225) ng·h/mL, respectively. In 21 patients receiving 40 mg/day afatinib therapy, the steady-state median (range) C_0_, C_max_, and AUC_0–24_ of afatinib were 30.4 (8.5–59.5) ng/mL, 47.9 (17.7–90.5) ng/mL, and 848 (289–1480) ng·h/mL, respectively. There were no significant differences in the C_0_, C_max_, and AUC_0–24_ of afatinib between patients receiving 30 and 40 mg/day doses. The interpatient variabilities (coefficients of variation) in afatinib C_0_ at 30 and 40 mg/day doses were 50.8% and 46.6%, respectively. The correlations between the AUC_0–24_ of afatinib and clinical characteristics of patients are shown in Table 2; however, there were no significant correlations.

### 3.3. Comparisons of Afatinib Pharmacokinetic Parameters or Clinical Characteristics between Patients with Grade 2 or Grade 0–1 Diarrhea

Comparisons of the pharmacokinetic parameters of afatinib or clinical characteristics of patients according to diarrhea grade (2 versus 0–1) are shown in Table 3. There were no patients with grade 3 diarrhea. The C_max_, C_0_, C_min_, AUC_0–24_, and AUC_6–24_ of afatinib in patients with grade 2 diarrhea were significantly higher than those in patients with grade 0–1 diarrhea; however, there were no significant differences in the clinical characteristics of patients between the two groups. In addition, there were no significant differences in the C_max_/C_min_ ratio and AUC_6–24_/AUC_0–24_ ratio, which is the enterohepatic circulation rate, of afatinib between the two groups (Table 3).

### 3.4. ROC Analysis and Kaplan–Meier Curves of Afatinib for the Incidence of Grade 2 Diarrhea

**Figure 2:**
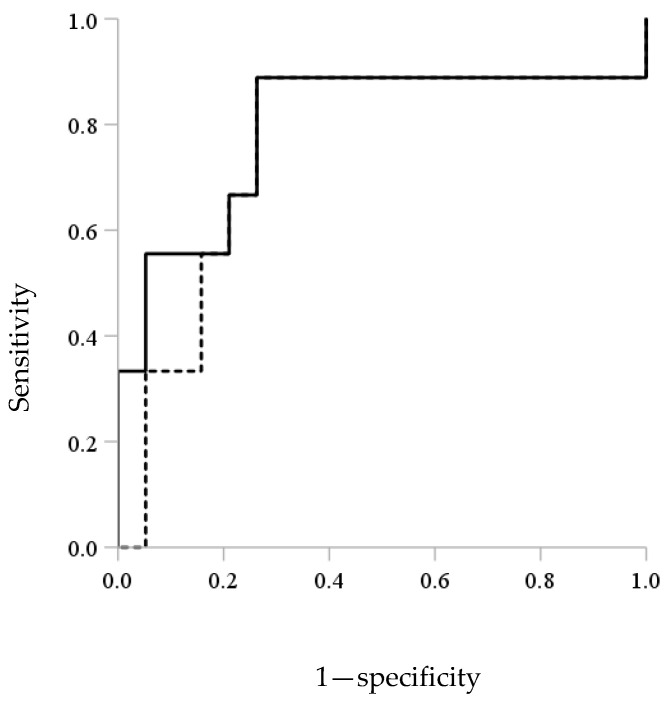
Receiver operator curve (ROC) analysis of the discrimination potential of AUC_0–24_ (solid line) and C_0_ (dashed line) of afatinib for the incidence of grade 2 diarrhea.

**Figure 3:**
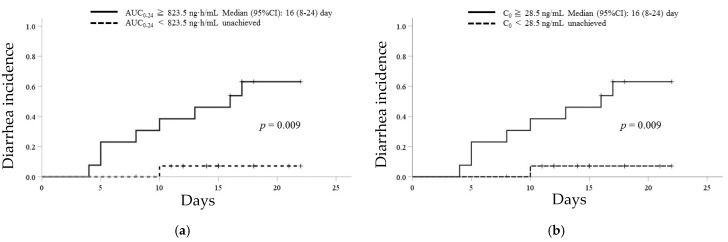
Kaplan–Meier analysis for time to the incidence of grade 2 diarrhea based on the cut-off values of AUC_0–24_ (823.5 ng·h/mL) and C_0_ (28.5 ng/mL) of afatinib. Kaplan–Meier curves for the incidence of grade 2 diarrhea in patients with (**a**) AUC_0–24_ of greater than or equal to 823.5 ng·h/mL (solid line) and less than 823.5 ng·h/mL (dotted line) and with (**b**) C_0_ of greater than or equal to 28.5 ng/mL (solid line) and less than 28.5 ng/mL (dotted line).

ROC analysis showed the discrimination potential of the AUC_0–24_ or C_0_ of afatinib for the incidence of grade 2 diarrhea (Figure 2). The areas under the ROC curves were 0.795 with the highest sensitivity (89%) and specificity (74%) at an AUC_0–24_ threshold of 823.5 ng·h/mL and 0.754 with the highest sensitivity (89%) and specificity (74%) at a C_0_ threshold of 28.5 ng/mL. Kaplan–Meier analyses for times to the incidence of grade 2 diarrhea based on these cut-off values of AUC_0–24_ (823.5 ng·h/mL) and C_0_ (28.5 ng/mL) of afatinib are shown in Figure 3. In patients with an AUC_0–24_ of greater than or equal to 823.5 ng·h/mL and a C_0_ of greater than or equal to 28.5 ng/mL, the median (95% CI) time to the incidence of grade 2 diarrhea was 16 (8–24) days. There was a statistically significant difference in the median time to the incidence of grade 2 diarrhea between patients with an AUC_0–24_ of greater than or equal to 823.5 ng·h/mL and less than 823.5 ng·h/mL or a C_0_ of greater than or equal to 28.5 ng/mL and less than 28.5 ng/mL (each *p* = 0.009, Figure 3).

### 3.5. Prediction Formulae to Estimate the Afatinib AUC_0–24_

The derived prediction formulae and *r*^2^ values for the estimation of the AUC_0–24_ of afatinib with a single point and with the best two-point combinations are shown in Table 4. Although a significant correlation between the AUC_0–24_ and C_0_ of afatinib was observed (*r^2^* = 0.761; *p* < 0.001), the predicted AUC_0–24_ of afatinib from the single point of C_6_ showed the highest correlation with the measured AUC_0–24_ (predicted AUC_0–24_ = 14.0 × C_6_ + 214.6, *r^2^* = 0.840; *p* < 0.001). In addition, the predicted AUC_0–24_ of afatinib from the two points of C_0_ and C_6_ showed the highest correlation with the measured AUC_0–24_ (predicted AUC_0–24_ = 10.6 × C_0_ + 9.1 × C_6_ + 135.4, *r^2^* = 0.911; *p* < 0.001).

## 4. Discussion

In the current study, the AUC_0–24_ and C_0_ of afatinib in patients with grade 2 diarrhea were significantly higher than those in patients with grade 0–1 diarrhea. We found that an afatinib AUC_0–24_ of greater than or equal to 823.5 ng·h/mL and a C_0_ of greater than or equal to 28.5 ng/mL may be used as cut-off values for the incidence of afatinib-induced grade 2 diarrhea. In addition, because afatinib C_0_ is related to AUC_0–24_, we could use C_0_ as a marker of therapeutic drug monitoring. Therefore, we monitored afatinib C_0_ on day 8 after the beginning of therapy to arrive at a steady state [14], and the daily dose of afatinib should be adjusted as an index with a cut-off value of 28.5 ng/mL. In the current study, the median time to the incidence of grade 2 diarrhea in the patients with a C_0_ of more than 28.5 ng/mL was 16 days. Therefore, we recommend monitoring the C_0_ of afatinib on day 8 after the beginning of afatinib therapy.

A higher afatinib C_0_ has been reported to be related to the severity of diarrhea [15]. In a previous study (the LUX-Lung trials) [15], the median C_0_ values of afatinib in patients with grade 2 or 1 diarrhea following the administration of 40 mg/day afatinib were reported to be 31.6 and 25.2 ng/mL, respectively. In addition, the median AUC_0–24_ values of afatinib in patients with grade 2 diarrhea in the LUX-Lung trials [14] and our current study were 1320 and 1225 ng·h/mL, respectively. Thus, the results obtained from the current clinical study were similar to the results of the LUX-Lung trials. To date, studies have suggested that female sex, low body weight, and reduced renal function are associated with higher afatinib exposure [23]. However, in an analysis using data pooled from seven clinical studies, the risk factors of afatinib-induced diarrhea were found to be older age, female sex, and low body weight (less than 45 kg) [24]. Therefore, patients with low body weight seem to be at risk of afatinib exposure-dependent diarrhea. Similar to the results of these previous studies [23,24], our current findings also showed that patients with lower body weight tended to have higher afatinib AUC_0–24_ (*p* = 0.070) and to develop grade 2 diarrhea (*p* = 0.085); however, the results were not significant. Therefore, afatinib therapy with a dose escalation strategy by therapeutic drug monitoring based on the target concentration of 28.5 ng/mL from a low dose of 20–30 mg/day for patients with a low body weight may be recommended to enable the administration of continuous treatment without interruption due to diarrhea.

Approximately 85% of afatinib is excreted into the bile as unchanged drug [15]. The biliary secretion of afatinib into the gut may directly induce diarrhea. Therefore, we evaluated the biliary secretion of afatinib using the AUC_6–24_/AUC_0–24_ ratio, which is the enterohepatic circulation rate. The results showed that there were no significant differences in the AUC_6–24_/AUC_0–24_ ratios of afatinib between patients with grade 2 or grade 0–1 diarrhea. Therefore, afatinib-induced diarrhea does not seem to be caused by the stimulation of the gut via the biliary excretion of afatinib. In addition, there were no significant differences in the C_max_/C_min_ ratio, which indicated the rate of absorption of afatinib, between patients with grade 2 and grade 0–1 diarrhea. Non-absorbed afatinib from the gut did not appear to contribute to diarrhea directly. By contrast, afatinib-induced diarrhea has been reported to be caused by the activation of apical membrane chloride (Cl^–^) channels in the intestinal epithelia rather than direct damage to the epithelium [25,26]. Therefore, further studies are necessary to determine the mechanisms mediating the onset of afatinib-induced diarrhea.

Overall, our current findings showed that afatinib exposure, including AUC_0–24_ and C_0_, was important for the prediction of grade 2 diarrhea onset.

To the best of our knowledge, no reports have validated an LSS for the prediction of the AUC_0–24_ of afatinib. Our results showed that C_6_ was the best single predictor of the AUC_0–24_ of afatinib, and an equation using samples measured at two specific points (C_0_ and C_6_) could best be used to approximate the AUC_0–24_ of afatinib. However, in outpatients, blood sampling for C_6_ after the administration of afatinib is difficult. Although the coefficient of determination (*r*^2^) between the predicted AUC_0–24_ of afatinib at the single point of C_0_ and the measured AUC_0–24_ was lower than that at the single point of C_6_ (*r*^2^ = 0.761 and 0.840, respectively), the 95% CI of the slopes and intercepts of the formulae obtained by bootstrap analysis also indicated acceptable accuracy and robustness for the prediction of AUC_0–24_ using the single point of C_0_. Therefore, the predicted AUC_0–24_ of afatinib with C_0_ alone was able to approximate the real AUC_0–24_. Consequently, the assessment of outpatients using an index of afatinib C_0_ with a cut-off value of 28.5 ng/mL is also possible. In the LUX-Lung 3 and 6 trials, median progression-free survival was similar between patients who received a reduced dose of afatinib and those who did not [27]. Similarly, in the real-world setting, time to treatment failure and time to progression did not change with the daily afatinib dose [28,29]. Furthermore, in a phase I study of afatinib plus bevacizumab, the recommended dose was set at 30 mg/day [30]. Therefore, it is important to adjust the dose of afatinib without hesitation because such adjustments are unlikely to affect efficacy. Our results can be used as an indicator for dose reduction owing to adverse effects.

Our results should be interpreted within the context of the study limitations. Unfortunately, in the current study, treatment with afatinib for patients with grade 3 diarrhea was halted before blood sampling for afatinib pharmacokinetics on day 15. Therefore, further studies are needed to determine the relationships between afatinib-induced grade 3 diarrhea and afatinib plasma concentrations. After beginning afatinib therapy, we may need to confirm the afatinib C_0_ at an early time on day 8 after the beginning of therapy to reach a steady state.

## 5. Conclusions

Afatinib AUC_0–24_ of greater than or equal to 823.5 ng·h/mL and C_0_ of greater than or equal to 28.5 ng/mL could be used as cut-off values for the incidence of afatinib-induced grade 2 diarrhea. In addition, because the afatinib C_0_ was related to AUC_0–24_, we could use C_0_ as a marker of therapeutic drug monitoring. Accordingly, we suggest monitoring the afatinib C_0_ on day 8 after the beginning of therapy to reach a steady state and adjusting the daily dose of afatinib as an index with a cut-off value of 28.5 ng/mL.

## Figures and Tables

**Table 1:** Demographic and clinical characteristics of patients prior to afatinib therapy.

Characteristics	Number or Values	
Total number	28	
Female:Male	13:15	
Age, years	67.4 ± 7.7	(51–86)
Body weight, kg	57.3 ± 9.4	(35.3–78.3)
Body surface area, m^2^	1.59 ± 0.16	(1.23–1.93)
Body mass index, kg/m^2^	22.7 ± 1.5	(19.8–25.8)
Laboratory test values		
White blood cell, ×10^3^/mm^3^	5.7 ± 1.4	(3.7–10.4)
Red blood cell, ×10^4^/mm^3^	422 ± 43	(342–498)
Hemoglobin, g/dL	12.6 ± 1.7	(8–15)
Platelets, ×10^4^/mm^3^	238 ± 59	(122–366)
Aspartate aminotransferase, IU/L	22.4 ± 5.4	(12–39)
Alanine aminotransferase, IU/L	16.9 ± 5.6	(8–30)
Alkaline phosphatase, IU/L	314 ± 218	(115–1336)
Lactate dehydrogenase, IU/L	219 ± 92	(135–601)
Serum albumin, g/dL	3.8 ± 0.4	(2.8–4.6)
Total bilirubin, mg/dL	0.5 ± 0.2	(0.3–1.1)
Serum creatinine, mg/dL	0.69 ± 0.21	(0.43–1.30)
eGFR, mL/min/1.73 m^2^	82.4 ± 21.4	(43.6–125.5)
Stage IV:IIIb:IIb	26:1:1	
Tumor history, adenocarcinoma:other	28:0	
EGFR mutation, exon 19 deletions:exon 21 L858R:other	16:7:5	
Initial dose, 30 mg:40 mg	7:21	
Diarrhea (grade 1:2): no diarrhea	23 (14:9):5	

Data are presented as number or mean ± standard deviation (range).

**Table 2:** Comparison and correlations of afatinib AUC_0–24_ with clinical characteristics of patients.

Characteristics	Median AUC_0__–__24_ (Range), ng·h/mL	*p*-Value
Female	848 (574–1480)	0.205
Male	753 (289–1366)	
	**Correlation Coefficient (r)**	***p*-Value**
Age	0.037	0.850
Body weight	−3.480	0.070
Body surface area	−2.540	0.192
BMI	−0.050	0.799
Laboratory test values		
White blood cell	0.115	0.561
Red blood cell	−0.293	0.130
Hemoglobin	−0.289	0.136
Platelets	−0.151	0.444
Aspartate aminotransferase	0.287	0.138
Alanine aminotransferase	−0.171	0.386
Alkaline phosphatase	−0.365	0.056
Lactate dehydrogenase	0.241	0.217
Serum albumin	−0.002	0.991
Total bilirubin	0.119	0.546
Serum creatinine	−0.070	0.724
eGFR	−0.107	0.587

AUC_0–24_, area under the plasma concentration–time curve from 0 to 24; eGFR, estimated glomerular filtration rate.

**Table 3:** Comparison of pharmacokinetics of afatinib and characteristics between patients with grades 2 and 0–1 diarrhea.

Parameters/Characteristics	Grade 2 Diarrhea	Grade 0–1 Diarrhea	*p*-Value
	Median (Quartile 1–Quartile 3)	Median (Quartile 1–Quartile 3)	
C_max_ (ng/mL)	78.0 (47.9–84.1)	38.9 (32.8–55.0)	0.017
C_0_ (ng/mL)	38.9 (33.1–42.0)	21.0 (15.0–29.8)	0.032
C_min_ (ng/mL)	28.1 (24.8–34.4)	16.5(14.5–25.0)	0.046
C_max_/C_min_ ratio	2.20 (1.90–2.70)	2.20 (1.75–2.45)	0.657
AUC_0__–__24_ (ng·h/mL)	1225 (891–1344)	666 (580–863)	0.013
AUC_6__–__24_ (ng·h/mL)	787 (672–950)	500 (424–592)	0.007
AUC_6__–__24_/AUC_0__–__24_ × 100 (%)	71.7 (67.8–73.3)	73.6 (69.7–75.8)	0.389
Daily dose, 30 mg:40 mg	1:8	6:13	0.249
Female:male	6:3	7:12	0.142
Age, years	65.0 (62.0–71.0)	67.0 (63.5–73.5)	0.693
Body weight, kg	50.5 (46.7–56.0)	56.2 (53.2–64.3)	0.085
Body surface area, m^2^	1.54 (1.41–1.57)	1.60 (1.54–1.73)	0.109
BMI, kg/m^2^	22.9 (22.8–23.3)	23.0 (21.3–23.4)	0.694
Laboratory test values			
White blood cell, ×10^3^/mm^3^	5.3 (4.0–6.9)	5.4 (4.1–6.0)	0.825
Red blood cell, ×10^4^/mm^3^	405 (384–430)	413 (372–455)	0.640
Hemoglobin, g/dL	12.1 (11.5–12.4)	12.5 (11.5–13.6)	0.403
Platelets, ×10^4^/mm^3^	21.4 (16.6–23.1)	23.7 (20.6–27.2)	0.210
Aspartate aminotransferase, IU/L	21 (18–22)	19 (18–28)	0.730
Alanine aminotransferase, IU/L	15 (11–28)	18 (14–25)	0.362
Alkaline phosphatase, IU/L	241 (211–419)	261 (226–290)	0.825
Lactate dehydrogenase, IU/L	174 (156–191)	176 (160–217)	0.980
Serum albumin, g/dL	3.4 (3.3–3.6)	3.7 (3.4–3.9)	0.311
Total bilirubin, mg/dL	0.5 (0.4–0.7)	0.5 (0.4–0.6)	0.439
Serum creatinine, mg/dL	0.67 (0.56–0.70)	0.74 (0.66–0.85)	0.110
eGFR, mL/min/1.73 m^2^	78.5 (62.3–97.3)	74.1 (65.9–82.6)	0.539

Data are presented as number or median (quartile 1–quartile 3). C_max_, maximum plasma concentration; C_0_, pre-dose concentration; C_min_, minimum plasma concentration; AUC_0–24_ and _6–24_, area under the plasma concentration–time curve from 0 to 24 h and 6 to 24 h, respectively; eGFR, estimated glomerular filtration rate.

**Table 4:** The prediction formulae derived using the multiple linear regression approach to estimate the AUC_0–24_ of afatinib.

Sampling Numbers	Sampling Time (h)	Prediction Formula for AUC_0–24_	Predicted versus Observed AUC_0–24_		Slope		Intercept 95% CI *	p *
			r^2^	*p*	95% CI *	p *		
One-point	0	22.3× C_0_ + 215.9	0.761	<0.001	17.7 to 28.6	0.001	85.5 to 331.2	0.005
	1	16.4 × C_1_ + 286.1	0.712	<0.001	12.2 to 21.8	0.001	143.7 to 411.0	0.001
	2	14.5 × C_2_ + 276.9	0.691	<0.001	10.6 to 19.7	0.001	110.3 to 433.1	0.012
	4	13.7 × C_4_ + 263.4	0.762	<0.001	10.5 to 18.0	0.001	112.1 to 410.0	0.007
	6	14.0 × C_6_ + 214.6	0.840	<0.001	11.4 to 17.3	0.001	81.7 to 334.6	0.004
	8	17.5 × C_8_ + 75.9	0.899	<0.001	15.6 to 20.1	0.001	−25.0 to 159.6	0.108
	12	23.8 × C_12_ + 11.9	0.916	<0.001	21.6 to 26.7	0.001	−75.2 to 84.7	0.770
Two-points †	0	10.6 × C_0_ + 9.1 × C_6_ + 135.4	0.911	<0.001	6.1 to 16.5	0.003	38.8 to 228.5	0.022
	6				5.6 to 12.3	0.001		

AUC_0–24_, area under the plasma concentration–time curve from 0 to 24 h; C*_n_*, plasma concentration at *n* h after afatinib administration. * Calculated using the bootstrap method. † Best sampling point.

## Data Availability

The data presented in this study are available on reasonable request.

## References

[1] Solca F., Dahl G., Zoephel A., Bader G., Sanderson M., Klein C., Kraemer O., Himmelsbach F., Haaksma E., Adolf G.R. (2012). Target binding properties and cellular activity of afatinib (BIBW 2992), an irreversible ErbB family blocker. J. Pharmacol. Exp. Ther..

[2] Machiels J.-P.H., Haddad R.I., Fayette J., Licitra L.F., Tahara M., Vermorken J.B., Clement P.M., Gauler T., Cupissol D., Grau J.J. (2015). Afatinib versus methotrexate as second-line treatment in patients with recurrent or metastatic squamous cell carcinoma of the head and neck progressing on or after platinum-based therapy (LUX-Head & Neck 1): An open-label, randomised phase 3 trial. Lancet Oncol..

[3] Yang J.C.-H., Shih J.-Y., Su W.-C., Hsia T.-C., Tsai C.-M., Ou S.-H.I., Yu C.-J., Chang G.-C., Ho C.-L., Sequist L.V. (2012). Afatinib for patients with lung adenocarcinoma and epidermal growth factor receptor mutations (LUX-Lung 2): A phase 2 trial. Lancet Oncol..

[4] Sequist L.V., Yang J.C.-H., Yamamoto N., O’Byrne K., Hirsh V., Mok T., Geater S.L., Orlov S., Tsai C.-M., Boyer M. (2013). Phase III study of afatinib or cisplatin plus pemetrexed in patients with metastatic lung adenocarcinoma with EGFR mutations. JCO.

[5] Katakami N., Atagi S., Goto K., Hida T., Horai T., Inoue A., Ichinose Y., Koboyashi K., Takeda K., Kiura K. (2013). LUX-Lung 4: A phase II trial of afatinib in patients with advanced non–small-cell lung cancer who progressed during prior treatment with erlotinib, gefitinib, or both. JCO.

[6] Wu Y.-L., Zhou C., Hu C.-P., Feng J., Lu S., Huang Y., Li W., Hou M., Shi J.H., Lee K.Y. (2014). Afatinib versus cisplatin plus gemcitabine for first-line treatment of Asian patients with advanced non-small-cell lung cancer harbouring EGFR mutations (LUX-Lung 6): An open-label, randomised phase 3 trial. Lancet Oncol..

[7] Park K., Tan E.-H., O’Byrne K., Zhang L., Boyer M., Mok T., Hirsh V., Yang J.C.-H., Lee K.H., Lu S. (2016). Afatinib versus gefitinib as first-line treatment of patients with EGFR mutation-positive non-small-cell lung cancer (LUX-Lung 7): A phase 2B, open-label, randomised controlled trial. Lancet Oncol..

[8] Tamura K., Nukiwa T., Gemma A., Yamamoto N., Mizushima M., Ochai K., Ikeda R., Azuma H., Nakanishi Y. (2019). Real-world treatment of over 1600 Japanese patients with EGFR mutation-positive non-small cell lung cancer with daily afatinib. Int. J. Clin. Oncol..

[9] Kim Y., Lee S.-H., Ahn J.S., Ahn M.-J., Park K., Sun J.-M. (2018). Efficacy and safety of afatinib for *EGFR*-mutant non-small cell lung cancer, compared with gefitinib or erlotinib. Cancer Res. Treat..

[10] Takeda M., Okamoto I., Nakagawa K. (2015). Pooled safety analysis of EGFR-TKI treatment for EGFR mutation-positive non-small cell lung cancer. Lung Cancer.

[11] Ding P.N., Lord S.J., Gebski V., Links M., Bray V., Gralla R.J., Yang J.C.-H., Lee C.K. (2017). Risk of treatment-related toxicities from EGFR tyrosine kinase inhibitors: A meta-analysis of clinical trials of gefitinib, erlotinib, and afatinib in advanced EGFR-mutated non–small cell lung cancer. J. Thoracic Oncol..

[12] Kato T., Yoshioka H., Okamoto I., Yokoyama A., Hida T., Seto T., Kiura K., Massey D., Seki Y., Yamamoto N. (2015). Afatinib versus cisplatin plus pemetrexed in Japanese patients with advanced non-small cell lung cancer harboring activating EGFR mutations: Subgroup analysis of LUX-Lung 3. Cancer Sci..

[13] Yang J.C.-H., Reguart N., Barinoff J., Köhler J., Uttenreuther-Fischer M., Stammberger U., O’Brien D., Wolf J., Cohen E.E. (2013). Diarrhea associated with afatinib: An oral ErbB family blocker. Expert Rev. Anticancer Ther..

[14] Wind S., Schmid M., Erhardt J., Goeldner R.-G., Stopfer P. (2013). Pharmacokinetics of afatinib, a selective irreversible ErbB family blocker, in patients with advanced solid tumours. Clin. Pharmacokinet..

[15] Wind S., Schnell D., Ebner T., Freiwald M., Stopfer P. (2017). Clinical pharmacokinetics and pharmacodynamics of afatinib. Clin. Pharmacokinet..

[16] Sato J., Morikawa N., Chiba R., Nihei S., Moriguchi S., Saito H., Yamauchi K., Kudo K. (2017). Case series on the association between blood levels and side effects of afatinib maleate. Cancer Chemother. Pharmacol..

[17] Hayashi H., Iihara H., Hirose C., Fukuda Y., Kitahora M., Kaito D., Yanase K., Endo J., Ohno Y., Suzuki A. (2019). Effects of pharmacokinetics-related genetic polymorphisms on the side effect profile of afatinib in patients with non-small cell lung cancer. Lung Cancer.

[18] Nakao K., Kobuchi S., Marutani S., Iwazaki A., Tamiya A., Isa S., Okishio K., Kanazu M., Tamiya M., Hirashima T. (2019). Population pharmacokinetics of afatinib and exposure-safety relationships in Japanese patients with EGFR mutation-positive non-small cell lung cancer. Sci. Rep..

[19] Miura M., Sato K., Miura H., Niioka T., Kobayashi H., Narita C., Ito H. (2014). A limited sampling strategy for estimation of the area under the plasma concentration–time curve of gefitinib. Ther. Drug Monitor..

[20] Yokota H., Sato K., Okuda Y., Kobayashi H., Takeda M., Asano M., Ito H., Miura M. (2017). Effects of histamine 2-receptor antagonists and proton pump inhibitors on the pharmacokinetics of gefitinib in patients with non-small-cell lung cancer. Clin. Lung Cancer.

[21] Nagahama M., Ozeki T., Suzuki A., Sugino K., Niioka T., Ito K., Miura M. (2019). Association of lenvatinib trough plasma concentrations with lenvatinib-induced toxicities in Japanese patients with thyroid cancer. Med. Oncol..

[22] Efron B., Tibshirani R.J. (1993). An introduction to the bootstrap. Monographs on Statistics and Applied Probability, No. 57. Chapman and Hall, London, 436 p. Monogr. Stat. Appl. Probab..

[23] Freiwald M., Schmid U., Fleury A., Wind S., Stopfer P., Staab A. (2014). Population pharmacokinetics of afatinib, an irreversible ErbB family blocker, in patients with various solid tumors. Cancer Chemother. Pharmacol..

[24] Hopkins A.M., Nguyen A.-M., Karapetis C.S., Rowland A., Sorich M.J. (2018). Risk factors for severe diarrhea with an afatinib treatment of non-small cell lung cancer: A pooled analysis of clinical trials. Cancers.

[25] Duan T., Cil O., Thiagarajah J.R., Verkman A.S. (2019). Intestinal epithelial potassium channels and CFTR chloride channels activated in ErbB tyrosine kinase inhibitor diarrhea. JCI Insight.

[26] Kim Y., Quach A., Das S., Barrett K.E. (2020). Potentiation of calcium-activated chloride secretion and barrier dysfunction may underlie EGF receptor tyrosine kinase inhibitor-induced diarrhea. Physiol. Rep..

[27] Yang J.C.-H., Sequist L.V., Zhou C., Schuler M., Geater S.L., Mok T., Hu C.-P., Yamamoto N., Feng J., O’Byrne K. (2016). Effect of dose adjustment on the safety and efficacy of afatinib for EGFR mutation-positive lung adenocarcinoma: Post hoc analyses of the randomized LUX-Lung 3 and 6 trials. Ann. Oncol..

[28] Halmos B., Tan E.-H., Soo R.A., Cadranel J., Lee M.K., Foucher P., Hsia T.-C., Hochmair M., Griesinger F., Hida T. (2019). Impact of afatinib dose modification on safety and effectiveness in patients with EGFR mutation-positive advanced NSCLC: Results from a global real-world study (RealGiDo). Lung Cancer.

[29] Liu C.-Y., Wang C.-L., Li S.-H., Hsu P.-C., Chen C.-H., Lin T.-Y., Kuo C.-H., Fang Y.-F., Ko H.-W., Yu C.-T. (2017). The efficacy of 40 mg versus dose de-escalation to less than 40 mg of afatinib (Giotrif) as the first-line therapy for patients with primary lung adenocarcinoma harboring favorable epidermal growth factor mutations. Oncotarget.

[30] Ko R., Shukuya T., Imamura C.K., Tokito T., Shimada N., Koyama R., Yamada K., Ishii H., Azuma K., Takahashi K. (2021). Phase I study of afatinib plus bevacizumab in patients with advanced non-squamous non-small cell lung cancer harboring EGFR mutations. Transl. Lung Cancer Res..

